# Dynamic neural network approach to targeted balance assessment of individuals with and without neurological disease during non-steady-state locomotion

**DOI:** 10.1186/s12984-019-0550-8

**Published:** 2019-07-12

**Authors:** Nathaniel T. Pickle, Staci M. Shearin, Nicholas P. Fey

**Affiliations:** 1 Department of Bioengineering, The University of Texas at Dallas, 800 W Campbell Rd, Richardson, TX 75080 USA; 2 Department of Physical Therapy, The University of Texas Southwestern Medical Center, 5323 Harry Hines Blvd, Dallas, TX 75390 USA; 3 Department of Physical Medicine and Rehabilitation, The University of Texas Southwestern Medical Center, 5323 Harry Hines Blvd, Dallas, TX 75390 USA

**Keywords:** Biomechanics, Rehabilitation, Gait, Balance, Wearable sensing, Sparse sensing, Machine learning, Parkinson’s disease

## Abstract

**Background:**

Clinical balance assessments often rely on functional tasks as a proxy for balance (e.g., Timed Up and Go). In contrast, analyses of balance in research settings incorporate quantitative biomechanical measurements (e.g., whole-body angular momentum, *H*) using motion capture techniques. Fully instrumenting patients in the clinic is not feasible, and thus it is desirable to estimate biomechanical quantities related to balance from measurements taken from a subset of the body segments. Machine learning algorithms are well-suited for this type of low- to high-dimensional mapping. Thus, our goal was to develop and test an artificial neural network that to predict segment contributions to whole-body angular momentum from linear acceleration and angular velocity signals (i.e., those typically available to wearable inertial measurement units, IMUs) taken from a sparse set of body segments.

**Methods:**

Optical motion capture data were collected from five able-bodied individuals and five individuals with Parkinson's disease (PD) walking on a non-steady-state locomotor circuit comprising stairs, ramps and changes of direction. Motion data were used to calculate angular momentum (i.e., “gold standard” output data) and body-segment linear acceleration and angular velocity data from local reference frames at the wrists, ankles and neck (i.e., network input). A dynamic nonlinear autoregressive neural network was trained using the able-bodied data (pooled across subjects). The neural network was tested on data from individuals with PD with noise added to simulate real-world IMU data.

**Results:**

Correlation coefficients of the predicted segment contributions to whole-body angular momentum with the gold standard data were 0.989 for able-bodied individuals and 0.987 for individuals with PD. Mean RMS errors were between 2 and 7% peak signal magnitude for all body segments during completion of the locomotor circuits.

**Conclusion:**

Our results suggest that estimating segment contributions to angular momentum from mechanical signals (linear acceleration, angular velocity) from a sparse set of body segments is a feasible method for assessing coordination of balance—even using a network trained on able-bodied data to assess individuals with neurological disease. These targeted estimates of segmental momenta could potentially be delivered to clinicians using a sparse sensor set (and likely in real-time) in order to enhance balance rehabilitation of people with PD.

## Background

Individuals with Parkinson’s disease (PD) commonly experience impaired balance. PD is a progressive neurological disorder that affects balance, with approximately 60% of people with PD reporting at least one fall in the past year [1]. In contrast with other conditions affecting balance through primarily mechanical changes, such as leg amputation, PD results in significant neural changes that lead to impairment of motor learning [2] and cognitive tasks such as set shifting (unconsciously shifting attention between tasks) [3–5]. Furthermore, changes in gait characteristics have been linked to early cognitive decline in people with PD [6]. Complex walking tasks may be particularly effective in revealing deficits in motor coordination and cognition that occur early in the disease progression because these tasks challenge both cognitive and motor function. People with PD exhibit difficulty planning and executing complex motor tasks [7] and struggle to perform concurrent cognitive and motor tasks [8]. In general, their neurological condition impairs “self-regulation,” which is an ability that is significantly challenged during complex tasks. However, there is also promising evidence that exercise, specifically involving challenging balance tasks, can help to slow disease progression [9], aid neural plasticity [10], and improve functional ability [11, 12].

Clinical balance assessments typically use proxy measures, such as walking speed or duration of single-leg standing, to assess balance. These quantities can be easily and rapidly assessed in clinical settings, but do not provide detailed quantitative data related to balance. In contrast to these clinical balance assessments, whole-body angular momentum (*H*) is a physics-based “Newtonian” quantity that gives direct insight into the mechanisms that relate to impaired balance. *H* is tightly regulated in level-ground walking in healthy individuals [13]. This regulation is achieved using muscles to generate an external moment about the body center of mass (COM) [13, 14]. The ability to regulate *H* is crucial for maintaining balance during dynamic tasks such as walking. This constraint can place large demands on the neuromuscular system, as a rapid muscle response is required in order to reduce *H* after tripping [15–17]. Also, an increased range of *H* is associated with impaired balance, as seen in elderly individuals with vestibular dysfunction [18], transtibial amputation [19–21] and stroke [22]. Furthermore, total *H* can be computed as the sum of each body segment’s angular momentum (i.e., superposition is valid). Analyses of the contributions of each segment to *H* can yield further insight into specific aspects of coordination of balance. This is particularly important in asymmetric pathologies, as other common clinical metrics such as gait velocity do not capture specific differences between the affected and unaffected sides of the body. Thus, providing clinicians with quantitative data related to balance may be extremely useful in both diagnosing and treating PD, especially early in the disease progression when deficits may be difficult to identify through visual assessment alone.

Despite the potential clinical value of measuring *H*, research studies that evaluate *H* typically rely on expensive motion capture systems, which can be either optical or wearable. Optical motion capture requires the often time-consuming process of placing reflective markers on anatomically relevant locations in order to track motion. Optical motion capture is also constrained to a limited capture volume (frequently occurring indoors), and post-processing can be laborious. In comparison, wearable sensing systems, e.g., those utilizing inertial measurement units (IMUs), are a relatively economical method for collecting quantitative movement data in clinical (and non-clinical) settings. Wearable systems have a number of advantages in comparison to research-grade optical motion capture systems. However, wearable sensors suffer from their own limitations, such as drift [23], which is an unbounded increase in position error caused by numerical integration of noisy and/or biased sensor data. In addition, the accuracy of low-cost sensors may not be sufficient for clinical intervention. Furthermore, a key requirement for any system intended for clinical use is that it must be easy to don and doff. Commercial wearable sensing systems typically require a sensor to be placed on each body segment, resulting in more than 10 sensors that must be affixed to the person. These systems are not practical for everyday clinical use. Yet despite these challenges, wearable IMU systems remain an appealing platform for deploying algorithms to assist medical providers in diagnosis and treatment of movement disorders because of their relatively low cost and accessibility.

One way to make wearable systems more clinically feasible is to develop algorithms that require fewer sensors. Reducing the number of sensors is desirable in order to increase ease of use, but doing so results in an underdetermined system because not all body segments are tracked. One way to address the problem of indeterminacy is to utilize machine learning to map from low-dimensional (sparse sensor set) to high-dimensional (whole-body biomechanics) spaces. Previous studies have utilized machine learning to estimate gait kinematics and kinetics from a sparse set of sensors. For example, two IMUs placed on the foot and shank were capable of predicting intra-subject hip, knee, and ankle joint angles with an absolute error of < 2.3° relative to the actual (optical motion capture) measurements [24]. Joint angle predictions within the same person were highly correlated with the actual values (r = 0.93–0.99) but much lower for inter-person predictions (r = 0.70–0.89). These results were achieved using a general regression neural network trained on data from eight subjects performing straight walking at their self-selected speed (30 gait cycles per person). Another prior study implemented an energy minimization algorithm that also enforced anthropometric constraints and coherence across frames [25], while utilizing six IMUs for tracking and four for validation. The algorithm was trained using data from four subjects performing a variety of motions including walking, running, punching, jumping and rotating their arms. This study achieved a mean orientation error of 13.24° (summed orientation errors of four validation IMUs) and mean position error of 3.9 cm (summed position error across 13 validation markers). Other researchers have approached the problem using a combination of optical motion capture markers and IMUs. A set of six IMUs and five reflective markers was able to achieve mean squared joint angle error of < 2° (average of each joint) and mean squared segment position error of 0.15 cm (average of each segment) [26]. This approach was unique in its use of a physics-based model of the body that enabled computation of inverse dynamics and ground reaction force prediction. The algorithm was trained using a database of 5000 representative poses. While these prior studies have achieved meaningful results, they focused primarily on kinematics (not on quantities directly related to balance) of relatively structured movements that are usually performed in isolation. Non-steady-state combinations of differing locomotor tasks have not been investigated. In addition, these machine learning approaches have not yet been tested in patient populations. Especially in the context of PD, fatigue occurs quickly [27], making it a challenge to collect the large volumes of data for varied movements that are needed for training an algorithm. It remains an open question whether machine learning algorithms are generalizable enough to be trained (or calibrated) on able-bodied individuals and implemented (or tested) on patients.

Motivated by the potential benefits to clinicians and patients, our objective was to develop a machine learning approach (i.e., artificial neural network, ANN) suitable for predicting *H* from a sparse set segmental data (linear acceleration and angular velocity) during relatively unstructured and non-steady-state locomotor scenarios. As a secondary objective, we aimed to train the network using only data from able-bodied individuals and test it on individuals with PD to assess the feasibility of this type of training paradigm for clinical applications. This preliminary study establishes proof-of-concept for the algorithm by testing it using virtual IMU data derived from optical motion capture data.

## Methods

**Fig. 1:**
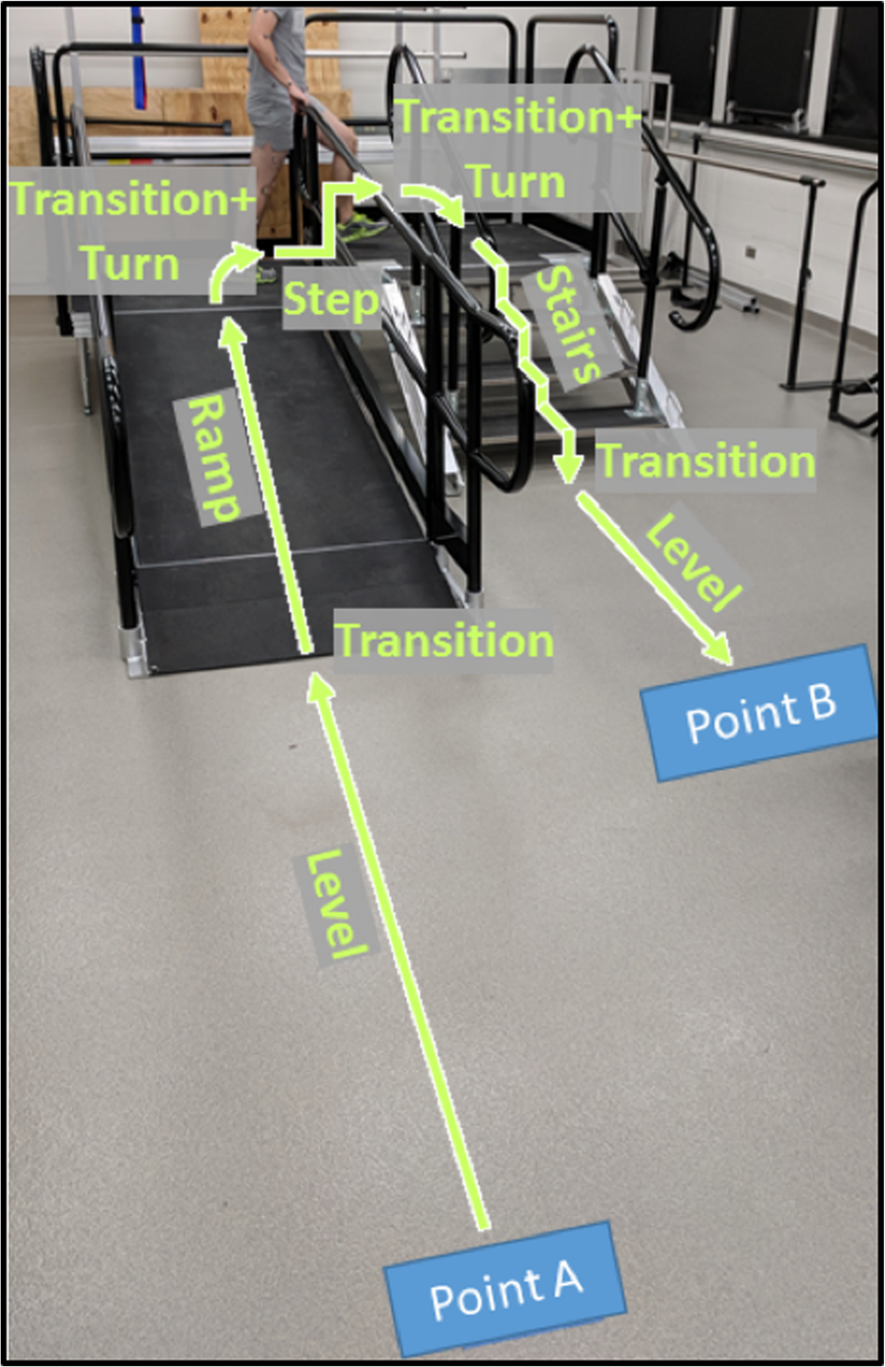
Diagram illustrating the locomotion tasks performed. Each participant began at Point A, went through the motions as shown, and stopped at Point B. In the next trial, the participant performed the tasks in the reverse order, starting at Point B and ending at Point A. The participants alternated leading with their left and right legs from each starting points. Kinematic data of the legs, arms and torso/pelvis were collected and processed for the entire movement

Five healthy individuals (4 M/1F, mean (SD) age 25.2 (2.5) years, height 1.75 (0.11) m, mass 66.8 (12.2) kg) and five individuals with early stage PD (Table 1) provided their written informed consent to participate in this protocol, which was approved by the Institutional Review Board at The University of Texas Southwestern Medical Center. Each participant with PD was assessed to determine their Hoehn and Yahr stage, which is a widely used measure to grade the severity of PD as related to unilateral or bilateral motor involvement and gait/balance changes. The scale has a possible score of 1 to 5. Stage 1 is unilateral motor impairment, stage 2 is bilateral motor impairment without balance difficulties, and stage 5 is confinement to a bed or wheelchair unless aided. All participants with PD in this study were Hoehn and Yahr stage 1 or 2 [28], did not experience freezing of gait during these experiments, did not have a deep brain stimulator implanted, and were medicated with their prescribed dosage at the time of testing. All participants were outfitted with 66 retroreflective markers placed on anatomically relevant locations to define 12 body segments. The 3D marker positions were tracked using a 10-camera optical motion capture system (Vicon, 100 Hz). Each participant walked on a circuit comprising a 4-step staircase with step height of 0.15 m and depth of 0.30 m, and a 2.5 m ramp inclined at 10° (Fig. 1). The step between the two platforms also had a height of 0.15 m. Participants performed sets of four walking trials in which they alternated starting from Point A and B as well as beginning the trial with their left and right leg. Able-bodied participants performed five sets while using the handrails, and five sets without using the handrails (total of 40 trials per person). Individuals with PD performed five sets (total of 20 trials per person) in which they were asked to walk at a comfortable pace, using the handrails as needed to ensure safety.

**Table 1:** Characteristics of Participants with Parkinson’s Disease

	Participant				
	1	2	3	4	5
Height (m)	1.72	1.72	1.72	1.80	1.72
Mass (kg)	94.8	54.4	82.6	92.5	63.5
Gender	M	F	F	M	F
Years since PD diagnosis	8	5	1.5	3	6
Weekly Exercise (min)	840	240	80	600	480
Falls Experienced	1	0	0	0	0
Dominant hand	R	R	L	R	R
Affected side	R	R	R	L	L
Hoehn and Yahr^a^	2	1	1	2	2

^a^Hoehn and Yahr stage determined by a licensed and practicing PT with over 10 years experience examining neurological gait disorders, and maintaining NCS physical therapy credentials. Height and mass were measured, other characteristics determined via self-report

### Data processing

**Fig. 2:**
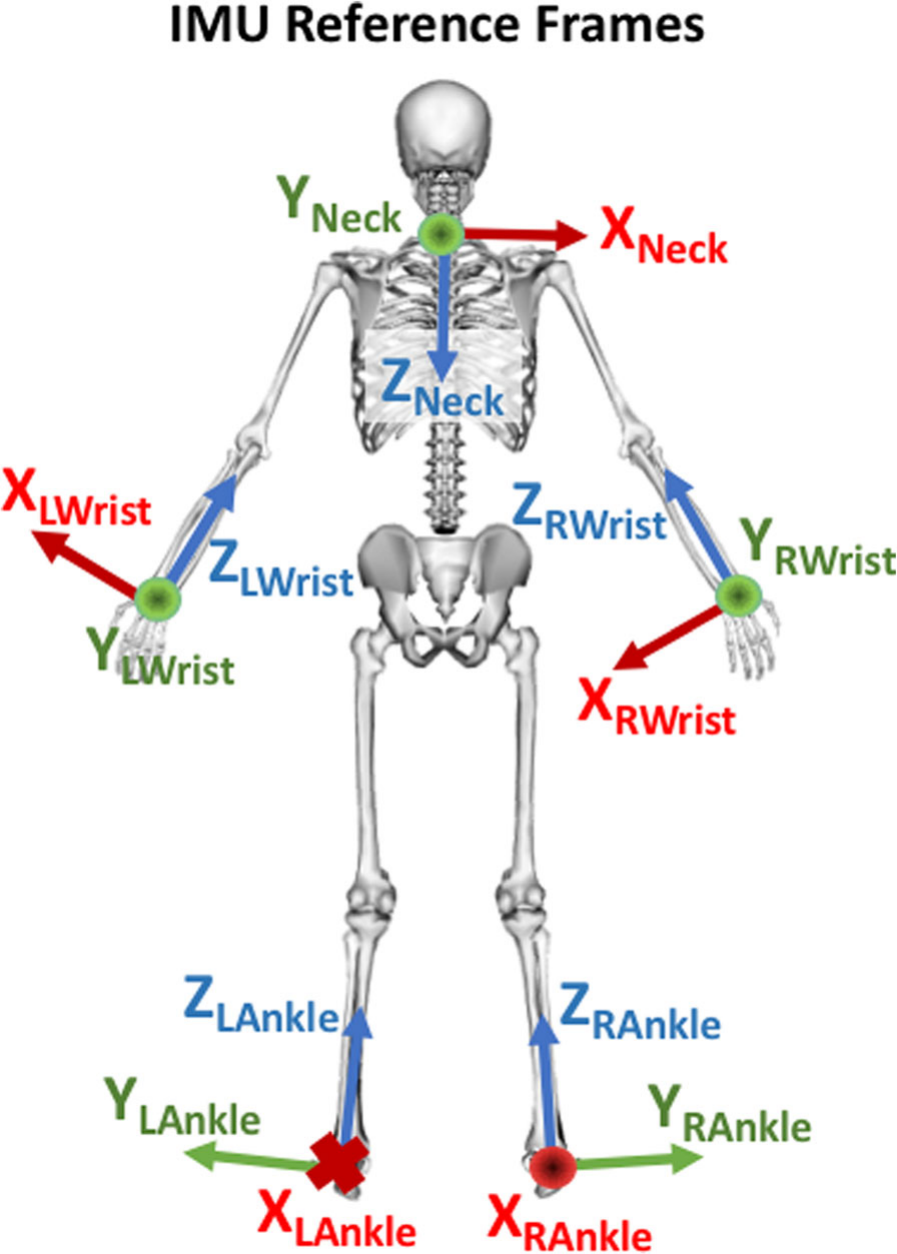
Diagram illustrating the locations of virtual IMU reference frames. “X” denotes into the page, “O” denotes out of the page

The marker trajectories were smoothed using a zero-lag low-pass 4th-order Butterworth filter with a cutoff frequency of 6 Hz. Dynamic models of each participant were created using Visual3D (C-Motion), with segment masses determined as a percentage of total body mass [29]. The contributions of segment *i* to *H* were computed using the equation

1 $$ {H}_i=\left({r}_i-{r}_{body}\right)\times {m}_i\left({v}_i-{v}_{body}\right)+{I}_i{\omega}_i $$

where *r*_*i*_ and *r*_*body*_ are the vector locations of segment *i* and the body COM, respectively, *v*_*i*_ and *v*_*body*_ are the velocity vectors of segment *i* and the body COM, respectively, *m*_*i*_ is the segment mass, *I*_*i*_ is the rotational inertia of the segment, and *ω*_*i*_ is the angular velocity of the segment. Contributions to *H* were expressed in a moving reference frame aligned with the body, similar to the path-dependent reference frame used by Gaffney et al. [30]. We defined the anterior direction (frontal plane) to be aligned with the anterior direction of the trunk segment coordinate system, the vertical direction (transverse plane) aligned with the lab vertical, and the mediolateral direction (sagittal plane) orthogonal to the anterior and vertical. Virtual IMUs were created by placing reference frames at the midpoint of the radial and ulnar wrist markers, the medial and lateral malleoli markers, and at the location of the 7th cervical vertebra (Fig. 2). The z-axes of the wrist virtual IMUs were directed toward the elbow joint centers and the y-axes toward the dorsal side of the hand. The z-axes of the ankle virtual IMUs were directed toward the knee joint centers, and the y-axes laterally. The z-axis of the neck virtual IMU was directed toward the midpoint of the posterior superior iliac spine (PSIS) markers, and the y-axis was directed posteriorly in a plane defined by the 7th cervical vertebra, PSIS midpoint, and a marker placed at the jugular notch. Gravity was modeled as a vertical acceleration of − 9.81 m/s in the laboratory reference frame, and the linear acceleration and angular velocity of the virtual IMUs were then transformed to be expressed in their local reference frames. Zero-mean pseudorandom noise with a Gaussian distribution was added to the simulated accelerometer (σ = 0.15 m/s) and gyroscope (σ = 0.005 rad/s) measurements during testing of the algorithm to simulate sensor noise. These noise amplitudes are more than twice as large as the noise specifications for the InvenSense MPU-9250 IMU used in low-cost sensors such as the TI SensorTag 6650, which would be suitable for clinical applications of our algorithm. Virtual IMU linear acceleration and angular velocity were filtered using a 5-frame moving average. The contributions to *H* of the arms (upper and lower arm), legs (thigh, shank, and foot) and trunk (torso and pelvis) were normalized by subject height and mass and filtered using a 5-frame moving average.

### Artificial neural network

**Fig. 3:**
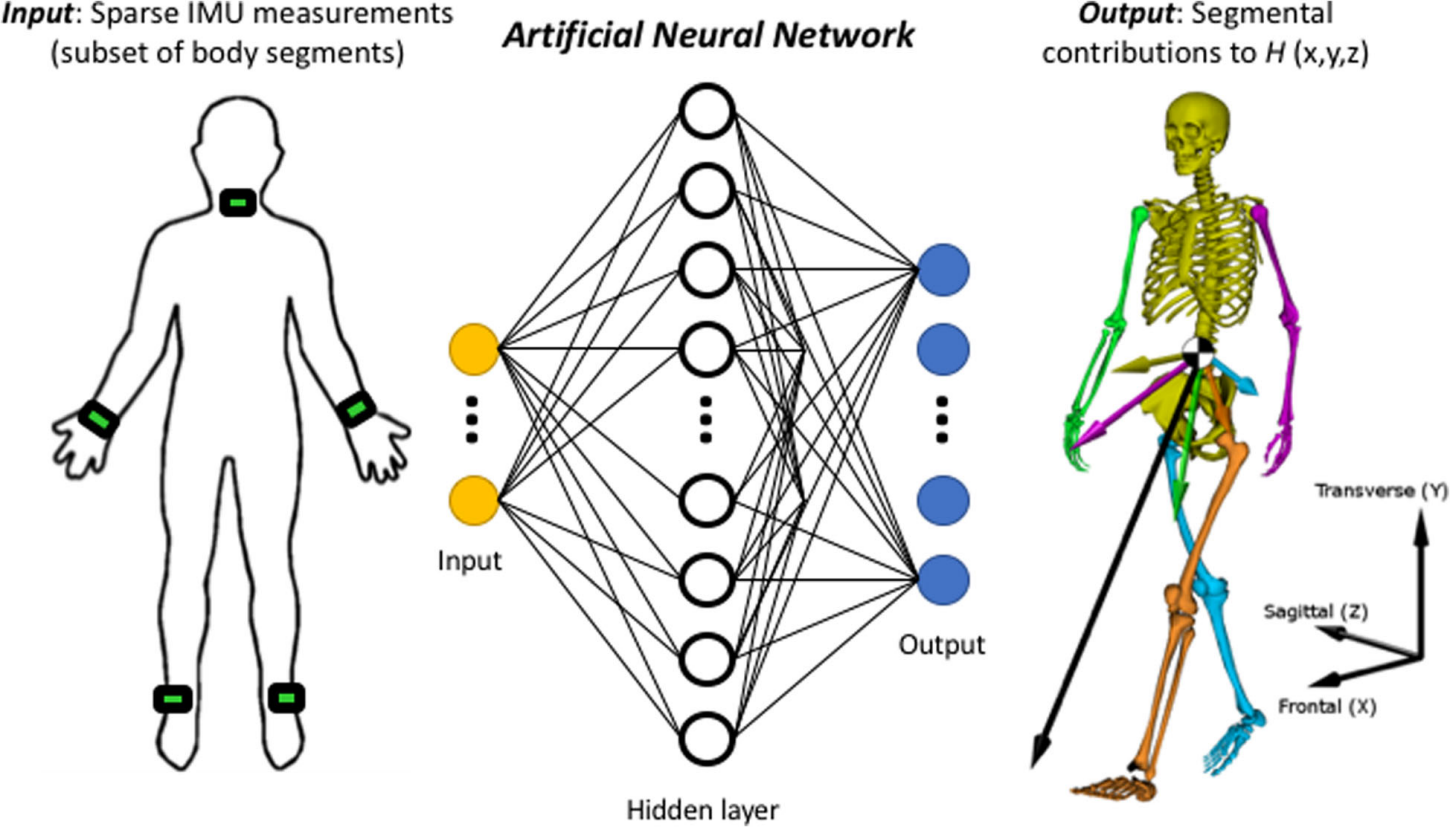
Diagram illustrating our machine learning approach using an artificial neural network (ANN). The ANN (center) maps from a sparse set of virtual inertial measurement units (IMUs, left) signals to segmental contributions to whole-body angular momentum (*H*) in three dimensions (right). Magnitude and direction of each segment’s contribution to *H* are shown as a vectors, as well as total *H* about the center-of-mass (black vector)

A dynamic nonlinear autoregressive neural network was selected and implemented using MATLAB (The Mathworks, Inc). The inputs to the network were 200 total sequences (40 trials for each of five able-bodied subjects) of 30-dimensional data (3D linear acceleration and angular velocity for five virtual IMUs). The network output was a 15-dimensional vector containing the 3D contributions to *H* from each of the five body segments (Fig. 3). The network comprised a single hidden layer of 30 neurons with sigmoid transfer functions and an output layer of 15 neurons with linear transfer functions. Delays were incorporated to provide the previous eight inputs and outputs to the network. The network was trained using the scaled conjugate gradient method [31]. The data were randomly partitioned; 70% used for training and 15% for validation (to avoid overfitting), and 15% for testing (assessing convergence). *Only able-bodied data* were used for training the network.

### Analysis

The segment contributions to *H* predicted by the neural network based on virtual IMU input data were compared to the actual contributions to *H* computed using optical motion capture data. We computed the correlation coefficient of the predicted segment contributions to *H* with the actual values. The root-mean-square (RMS) error between the predicted and actual values were computed and normalized by the peak signal magnitude to facilitate comparison of relative error magnitude in each body segment and within each plane of motion.

## Results

**Fig. 4:**
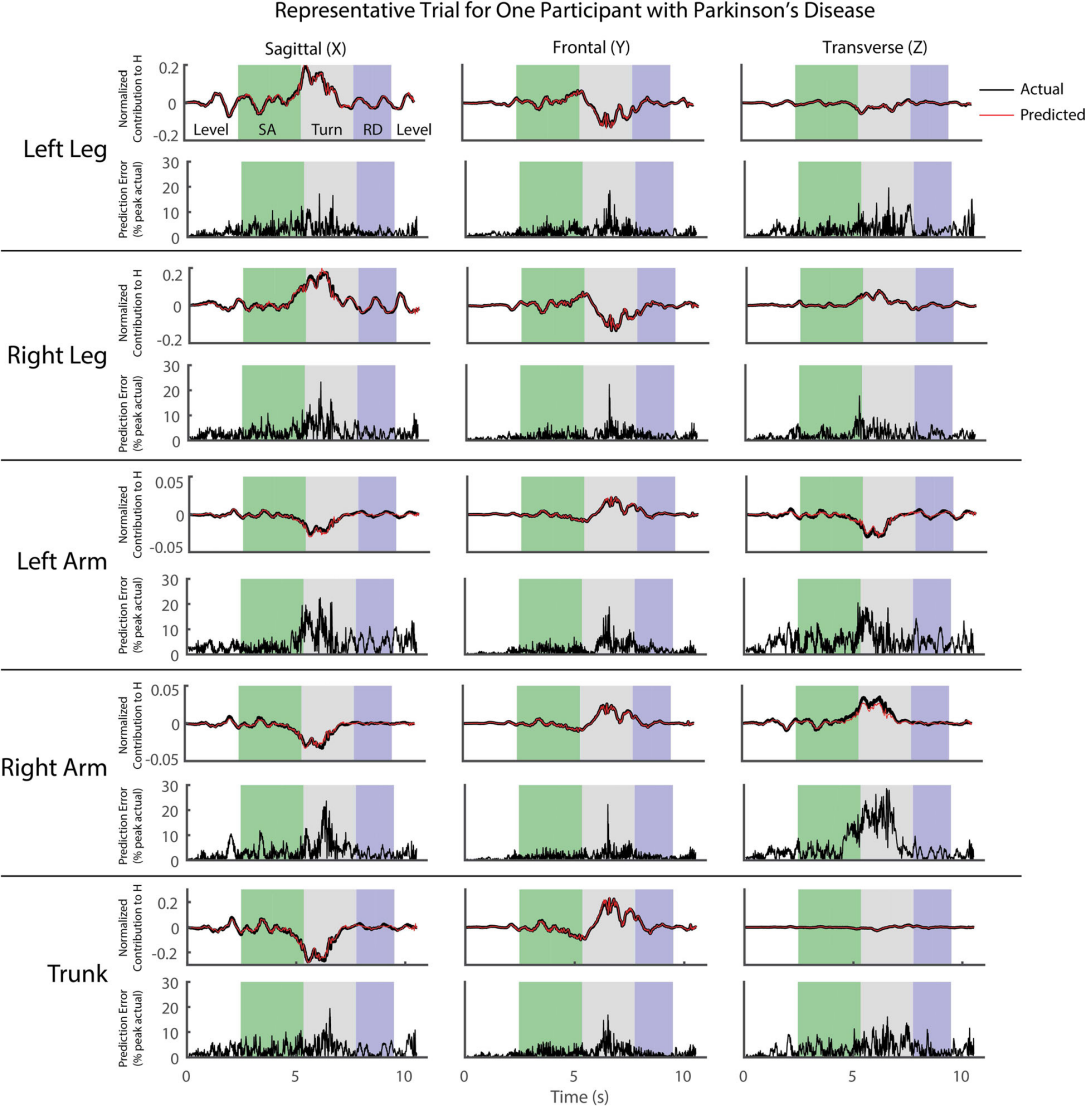
Representative trial for a subject with Parkinson’s disease. The contributions of each segment to whole-body angular momentum (*H*) are given. For each segment, the top row of plots shows the actual (black line) and predicted (red line) contributions to *H*, normalized by body height and mass (note the different scales), and the bottom row shows the prediction error normalized to a percentage of the peak actual signal magnitude. Shaded regions indicate level ground walking (white), stair ascent (SA, green), turn+step (gray), and ramp descent (RD, purple)

There were strong correlations between the predicted and actual (optical motion capture-based) segment contributions to *H* when tested on both able-bodied (r = 0.989) and PD (r = 0.987) individuals. The mean RMS errors ranged from approximately 1.8 to 6.4% peak signal magnitude (Table 2). The errors did not appear to be greater or smaller in any segment relative to the others (Fig. 4). However, for both able-bodied and PD participants, the errors appeared to be smallest in the frontal plane. The mean RMS errors for each signal also appeared to be somewhat larger in individuals with PD compared to the corresponding signal in able-bodied individuals, which was a somewhat expected result given PD data were not used to train the network.

**Table 2:** Mean (SD) RMS Error in Segment Contributions to Whole-Body Angular Momentum (% of Peak Signal Magnitude)

	Control			Parkinson’s		
	Sagittal	Frontal	Transverse	Sagittal	Frontal	Transverse
Left Leg	3.63 (1.00)	1.87 (0.44)	3.68 (1.49)	4.72 (1.55)	2.30 (0.69)	4.76 (1.90)
Right Leg	3.53 (1.02)	1.82 (0.40)	3.61 (1.48)	4.58 (1.53)	2.15 (0.60)	4.46 (1.87)
Left Arm	3.77 (1.15)	1.88 (0.43)	4.47 (1.51)	5.84 (2.42)	2.66 (1.18)	6.39 (1.68)
Right Arm	4.41 (1.30)	1.96 (0.49)	4.32 (1.33)	5.84 (2.28)	2.43 (0.93)	5.93 (1.72)
Trunk	3.32 (0.84)	1.79 (0.44)	4.26 (1.73)	4.40 (1.14)	2.00 (0.57)	4.75 (1.99)

## Discussion

The aim of this study was to develop and test a machine learning approach (i.e., artificial neural network) to predict segmental contributions to *H* from a sparse set of mechanical signals during relatively unstructured and non-steady-state locomotor scenarios. Our motivation was to develop an algorithm that can be implemented in wearable IMU sensors, which would be beneficial in a number of clinical applications. Our algorithm achieved accurate predictions (RMS error < 7% peak signal magnitude) of segment contributions to *H* when trained and tested with optical motion capture data. Notably, the network was trained using *only able-bodied data*, and was able to predict segment contributions to *H* with similar accuracy in individuals with and without PD. The correlation of model predictions with target values was very high (r > 0.98 for both groups) and the RMS errors were all less than 7% of the peak signal magnitude on average. These results appear to exceed performance of prior studies that found inter-subject correlations between 0.70 and 0.89 [24], and were limited to studying steady-state walking.

Our algorithm was similar to Findlow et al. [24], using an ANN to map directly from IMU sensor measurements to a biomechanical quantity of interest, but with a couple of notable differences. First, our experimental protocol included a wide variety of walking tasks: level ground, ramp ascent/descent, stair ascent/descent, turns, and transitions between each of these tasks. In addition, we collected 200 total trials, each lasting approximately 10–12 s. This large amount of rich biomechanical data likely provided an advantage over analyzing a smaller number of level-walking gait cycles. A second difference is that we used the ANN to predict segment contributions to *H*, which have direct connections (i.e., a Newtonian relationship) to regulation of balance. These data could be used to augment clinical treatment of balance by providing clinicians with quantitative (i.e., actionable) data that can be used to objectively identify balance deficits as well as track performance over time. Furthermore, ANN’s are computationally fast, enabling feedback to be provided quickly, potentially in real-time. Implementation of the algorithm used in this study would require only a moving average filter on each input signal, and then multiplying the inputs and prior outputs (current value and eight prior values of inputs and outputs) by the ANN weights. Although training and recalibration of the ANN is computationally expensive, training can be performed offline, outside of patient sessions, and as we have demonstrated may rely solely on able-bodied data.

There are several important limitations to this study. While our approach of directly estimating contributions to *H* from linear acceleration and angular velocity allows us to avoid issues related to drift and achieve computational efficiency, it also precludes us from computing joint angles and body segment positions. Obtaining position and orientation data could be useful for applications such as visualization of data for graphical feedback to either the patient or clinician. Methods such as those used by von Marcard et al. [25] or Andrews et al. [26] incorporate an underlying human body model, and could be extended to compute *H* in addition to kinematics. Another limitation of this study is that we used simulated IMU sensor data and have not yet tested the algorithm in physical IMU sensors. Although we modeled sensor noise in our simulated test data, other real-world issues such as sensor placement may affect algorithm performance, and should be investigated in future work. While our algorithm predicted segment contributions to *H* with RMS error < 7% peak signal magnitude, it remains unclear whether this accuracy is high enough for clinical treatment. To our knowledge, segment contributions to *H* have not been used to provide biofeedback during walking, and therefore it is not clear what level of accuracy is needed in order to identify deviations in gait patterns. In addition, more work is needed to better understand segment contributions to *H* in individuals with PD, especially given the variability in severity, affected regions of the body, and type of impairment (e.g., bradykinesia, dyskinesia, etc.) across individuals with PD. This study included a small sample of participants who exhibited mild symptoms and were medicated during the experiment, and we did not attempt to simulate pathology-related phenomena such as tremor when training the algorithm. Algorithm performance may worsen in individuals with more severe symptoms or require the network to be trained on patient data rather than the able-bodied training data used in this study.

In this study, we elected to use complex motor tasks that challenge neural task planning and coordination of locomotion. However, because PD affects cognitive processes such as set shifting, it may be useful to also investigate the effects of dual-tasking on coordination of balance. Assessing cognition would help further evaluate the link between cognitive and motor impairments. In addition, including a concurrent cognitive task with the walking task could further challenge the person’s ability to plan and execute the complex motor task. Future investigations highlighting these links between cognitive and motor deficits would be useful.

## Conclusion

Using machine learning techniques such as artificial neural networks to predict segment contributions to *H* from mechanical signals (linear acceleration and angular velocity) from only five body segments (left/right forearm, left/right shank, and torso) is feasible. Furthermore, our results suggest that these systems can be trained using only able-bodied data, and still demonstrate high accuracy when tested on people with early-stage PD. Finally, a single network can be used across a rich set of relatively unstructured and non-steady-state locomotor tasks. These results suggest the feasibility of an economical system capable of quantitative assessment of balance that can be used outside of the laboratory and in clinical environments.

## Data Availability

The datasets used and/or analyzed during the current study are available from the corresponding author on reasonable request.
